# Ketoacidosis at diagnosis of type 1 diabetes in children and adolescents: frequency and clinical characteristics

**DOI:** 10.1186/2251-6581-12-47

**Published:** 2013-12-19

**Authors:** Alphonsus N Onyiriuka, Emeka Ifebi

**Affiliations:** Department of Child Health, University of Benin Teaching Hospital, PMB 1111, Benin City, Nigeria

**Keywords:** Children, Characteristics, Ketoacidosis, Type 1 diabetes mellitus, Prevalence

## Abstract

**Background:**

Diabetic ketoacidosis (DKA) is a potentially life-threatening acute complication of type 1 diabetes mellitus (T1DM). Although the frequency of DKA as first manifestation of T1DM is higher in developing compared developed countries, there is paucity of information on its characteristics in developing countries.

**Methods:**

This retrospective study determined the frequency of ketoacidosis at diagnosis of new-onset type 1 diabetes and described the clinical characteristics of the patients seen between 1996 and 2011 by auditing the hospital records of all cases. The diagnosis of diabetic ketoacidosis (DKA) was based on the presence of hyperglycaemia (blood glucose > 11 mmol/L), acidosis (serum bicarbonate < 15 mmol/L) and ketonuria (urine ketone ≥1+).

**Results:**

At diagnosis of new-onset type 1 diabetes mellitus, three-quarter (77.1%) of the children and adolescents presented with DKA. Comparing the frequency of DKA during the initial 8 years (1996–2003) with the later 8 years (2004–2011), it was 81.8% vs 73.1%; p > 005. The frequency has not shown any significant declined over a 16-year period. The frequency of re-admission in ketoacidosis was 24.3%.

**Conclusion:**

Three-quarter of children and adolescents present with DKA as first manifestation of T1DM with no significant decline in frequency over a 16-year period in our hospital.

**Electronic supplementary material:**

The online version of this article (doi:10.1186/2251-6581-12-47) contains supplementary material, which is available to authorized users.

## Introduction

Diabetic ketoacidosis (DKA) is a potentially life-threatening acute complication of type 1 diabetes mellitus (T1DM), characterized by a biochemical triad of hyperglycaemia, ketonaemia (ketonuria) and acidaemia.^1^ DKA is caused by a decrease in effective circulating insulin associated with elevations in counterregulatory hormones [1, 2].

The likelihood of ketoacidosis occurring at the onset of diabetes varies considerably (between 15% and 67%) from one country to another [3]. Studies have shown that in parts of the world where the prevalence of T1DM is low, symptoms of diabetes may be less familiar to medical practitioners, as a consequence more DKA occurs as the initial presentation of diabetes [4]. The same is true where access to medical care is limited. Observational studies from African countries suggest that the frequency is high but the exact figures are scarce. However, the report of a few studies from Africa indicated that the frequency varied from 80% to 88% [5–7]. The reason for DKA at onset of newly-diagnosed diabetes is multifactorial [8]. The management of DKA, in itself, imposes a significant economic burden on the patient and his family, particularly in developing countries [9, 10]. DKA is the most frequent cause of diabetes-related death in children with the mortality rate ranging between 6% and 24% in developing countries [11, 12].

Understanding factors associated with DKA has the potential of improving our knowledge of the disease, enhancing the development of patient, professional- and population-based interventions to reduce the proportion of children whose first presentation is DKA. The purpose of the present study was to determine the frequency of DKA at diagnosis of new cases of T1DM and to describe the clinical characteristics of ketoacidosis among these patients.

## Methods

In this retrospective study, the case records of all children and adolescents with type 1 diabetes mellitus complicated by diabetic ketoacidosis seen in the Department of Child Health, University of Benin Teaching Hospital (UBTH), between 1996 and 2011 were retrieved and audited. Information extracted included age, sex, presenting features, laboratory findings, parents educational attainment and occupation, fluid/electrolyte/insulin management, complications, and outcome of the patient. The socio-economic status of the patients’ parents was determined using the criteria suggested by Ogunlesi et al. [13]. This was analyzed via combining the highest educational attainment, occupation and income of the parents (based on the mean income of each educational qualification and occupation). In this way, the subjects were categorized into socioeconomic classes: class I (high), class II (middle) and class III (low). The study design was approved by the relevant hospital (UBTH) authority. The current criteria for the diagnosis of DKA published by the International Society for Paediatric and Adolescent Diabetes (ISPAD) is blood glucose concentration greater than 11 mmol/L, blood pH less than 7.3 (serum bicarbonate level < 15 mmol/L) and ketonuria [14]. The severity of DKA is classified based on the degree of acidosis into mild, venous pH 7.2-7.3 (bicarbonate 15–18 mmol/L); moderate, pH 7.1- <7.2 (bicarbonate 10–14 mmol/L); and severe, pH < 7.1 (bicarbonate < 10 mmol/L) [2, 14]. Outcome was measured in terms of death or survival. Descriptive statistics such as frequencies, means, ratios, standard deviations, confidence intervals, percentages were used to describe all the variables. The significance of differences in proportion was assessed with Z-test while the differences in means was assessed with Student *t* test with p-value set at <0.05.

### Consent

Informed consent was obtained from the patients/their parents for publication of this report.

## Results

Among a total of 48 patients with Type 1 diabetes mellitus (T1DM) who were hospitalized during the 16-year period under review, 37(77.1%), 95% Confidence Interval, CI = 76.2-78.0, presented with diabetic ketoacidosis (DKA) at first diagnosis of diabetes. Of these 37, 15(40.5%) were males while 22(59.5%) were females, giving a male-to-female ratio of 1:1.5. Comparing the frequency of DKA among newly diagnosed diabetes during the initial 8 years (1996–2003) with during the later 8 years (2004–2011), it was 81.8% (18/22) vs 73.1% (19/26), representing a decline of 8.7%; Z-statistic = 0.727 p > 0.05. The mean age at presentation in relation to gender was for boys 11.4 ± 3.9 years, 95% CI = 9.4-13.4 and for girls 13.2 ± 1.8 years, 95% CI = 12.4-14.0; t = 1.671 p > 0.05. When both sexes were combined, the mean age at presentation was 12.7 ± 2.6 years, 95% CI = 11.9-13.5. The age and gender distribution of patients with DKA are displayed in Table 1.

**Table 1:** **Distribution of age and gender of patients with diabetic ketoacidosis**

Age (years)	Gender		
	Male	Female	Both sexes
	No (%)	No (%)	No (%)
Below 10	2 (13.3)	0 (0.0)	2 (5.4)
10–12	2 (13.3)	6 (27.3)	8 (21.6)
13–15	8 (53.3)	14 (63.6)	22 (59.5)
Above 15	3 (20.1)	2 (9.1)	5 (13.5)
Total	15 (100.0)	22 (100.0)	37 (100.0)

**Figure 1:**
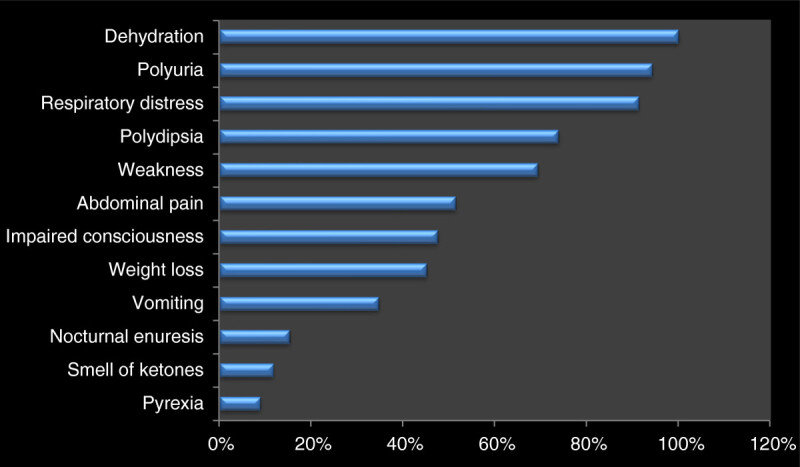
**Frequency of signs and symptoms among 37 patients with diabetic ketoacidosis.**

During the period under review, seven (19.8%; 5 girls and 2 boys) of the 37 patients who were earlier registered and treated for DKA were hospitalized for the second time in ketoacidosis, giving a female-to-male re-admission ratio of 2.5:1. Omission of insulin was the precipitating factor of DKA in 4 (57.1%) of the 7 patients who were previously diagnosed and were being followed up for T1DM. These four cases who omitted insulin were because of proclaimed cure at spiritual healing homes. Other clinical characteristics of the patients are depicted in Table 2. The serum electrolyte profile at the point of admission revealed that 27(73.0%) out of the 37 cases had moderate DKA. The remaining 10(27.0%) had severe DKA. Over half (52.3%)of the families of the patients with DKA at initial presentation were of the middle social class while 11.2% were of the high social class and 36.5% were of the low social class. Out of 37 cases of newly-diagnosed T1DM manifesting with DKA, 69.4% had symptoms for 2–3 weeks before presentation and 29.2% had had at least one medical consultation during the period in a private clinic. Two (8.3%) had more than one medical consultations during the same period. The distribution of duration of symptoms before presentation was as follows: less than 2 weeks 10.9%; 2–4 weeks 46.4%; 5–7 weeks 27.3%; and above 7 weeks in 15.4%. The frequency of the presenting symptoms and signs is displayed in Figure 1. Eight (21.6%) of the mothers whose children presented with DKA admitted they did not know that diabetes mellitus can occur in children. The frequency of a positive family history of diabetes mellitus was 8.1% and this involved two mothers and a maternal grandmothers.

**Table 2:** **Clinical characteristics of patients presenting with DKA at initial diagnosis of T1DM**

Clinical characteristics	Mean values	95% CI
Mean duration of symptoms	2.3 ± 1.2 weeks	1.9-2.7
Mean blood glucose at point of hospitalization	27.8 ± 10.6 mmol/L	24.4-31.2
Mean serum potassium	3.1 ± 1.6 mmol/L	2.6-3.6
Mean duration of fluid therapy	48.7 ± 10.3 hours	45.4-52.0
Mean time of switching from insulin infusion to SC insulin	51.6 ± 12.4 hours	47.6-55.6
Discharge against medical advice	1 (2.7%)	
Outcome of DKA (death or survival)	No death	

*SC = Subcutaneous; CI = Confidence interval.*

Two (9.1%) of the 22 girls with DKA had delayed pubertal maturation; Tanner Stage II at the ages of 15 and 16 years respectively. None of the two has attained menarche at the ages of 16 and 17 years respectively. Their weights and body mass indices were 29 Kg and 30 Kg; 16.0 Kg/m^2^ and 17.4 Kg/m^2^ respectively (both BMI below the 5th percentile). One of the two girls had vaginal candidiasis.

In all the patients, therapy was started with normal saline and a continuous infusion of regular insulin at a rate of 0.1 Units/kg/hour. Capillary blood glucose and serum electrolytes were monitored 2 hourly and 12 hourly respectively. As shown in Table 3, hypoglycaemia was the most frequent complication encountered during the management of DKA. No death was recorded.

**Table 3:** **Frequency of complications encountered in patients admitted for DKA**

Complications	Number	Percent
Hypoglycaemia (blood glucose < 3.3 mmol/L)	6	16.2
Hyponatraemia (serum sodium < 135 mmol/L)	3	8.1
Hypokalaemia (serum potassium < 3 mmol/L)	2	5.4
Hypocalcaemia (serum calcium < 2.2 mmol/L)	1	2.7
Hyperkalaemia (serum potassium > 5.5 mmol/L)	1	2.7

## Discussion

Slightly over three-quarter of the children and adolescents in the present study manifested with DKA at first diagnosis of diabetes. This is not surprising as a similar high prevalence rate has been reported in a previous study in Nigeria [15]. The same is true of reports from Iran and the United Arab Emirates [16, 17]. However, some studies [18, 19] have reported lower prevalence rates, all reflecting the well known wide geographic variation in frequency of DKA at onset of paediatric diabetes mellitus [2, 4]. The differing prevalence rates might be explained by differences in study population, the background prevalence of diabetes in the given population, presence or absence of family history of T1DM, family socioeconomic status, delayed diagnosis and treatment as well as the definition of DKA used in the particular study. All these factors have been reported to influence the prevalence of DKA at onset of type 1 diabetes [18–20]. The relatively higher mean age (12.7 years) of our patients may contribute to the observed higher prevalence. This view is supported by a report from Finland which indicated a higher prevalence among patients aged 10–14 years compared to those aged 5–9 years [21]. Secular changes in frequency of DKA at onset of T1DM has equally been reported [21]. The clinical implication of DKA as first presentation T1DM may be viewed from two perspectives as proposed by Neu et al. [8]. First, as a reflection of a delayed diagnosis and treatment or as an aggressive form of diabetes. Either way, it represents a poor prognostic factor. For instance, DKA in newly diagnosed diabetes has been linked to a poorer glycaemic control, [20, 22] less residual β-cell function (up to two years) after diagnosis, [23, 24] and lower frequency of remission [25, 26].

Data from the present study indicate that over the years, the frequency of DKA at diagnosis of new-onset T1DM has not shown any significant decline when the frequency during the initial 8 years (1996–2003) of the 16-year review was compared with the frequency in the later 8 years (2004–2011). This finding is consistent with report of Bui et al. [27]. In contrast, a study in Northern Finland reported a significant decline in frequency (18.9 versus 29.5%) of DKA at first presentation of TIDM over the later 10 years of a 20-year review [22]. The explanation is that the high background prevalence of diabetes mellitus in Finland makes the recognition of the disease easier by both the public and the physician. Increased level of awareness of early symptoms of diabetes among the populace and greater medical alertness to the occurrence of TIDM among the physicians will be rewarding. This view is supported by two different findings in the present study. First, nearly one-third of the patients who had symptoms for 2–3 weeks before presentation in DKA had at least one medical consultation during that period in a private clinic, suggesting missed diagnosis and, therefore, a window for intervention. Secondly, over one-fifth of the mothers whose children presented in DKA thought that diabetes mellitus was a disease exclusive to the adult population, suggesting the need to increase general public awareness of the symptoms of diabetes mellitus.

Consistent with previous reports, [17, 18] there was a female preponderance among DKA patients in the present study. This finding may be explained by the hormonal changes which accompany puberty, particularly the elevation in the serum levels of some counter-regulatory hormones, such as growth hormone and oestrogen. These hormonal changes differ between boys and girls. For instance, the level of oestrogen is by far higher in girls than boys at puberty. In addition, cytokines such as interleukin 1 (IL 1), interleukin 6 (IL 6) and tumour necrosis factor (TNF) -alpha produced during stress antagonize the effects of insulin promoting the occurrence of DKA [28]. It is possible that cytokine-response pattern to stress may differ between boys and girls, precipitating DKA in girls with elevated counter-regulatory. As noted earlier, the basic pathogenesis of DKA is a decrease in the effective circulating insulin associated with an elevation in the serum counter-regulatory hormones [1, 2].

The mean age at presentation in the present study was significantly higher than that reported from some countries: Iran; [17] Saudi Arabia; [18] and Spain, [29] suggesting that childhood T1DM presents at a relatively older age in Nigeria. The youngest child in our series was 5 years old. The reason for this difference is not clear. It might be related to racial variations.

In the present study, over one-quarter had severe form of DKA. This is in agreement with the frequency of severe DKA reported from Kuwait [19]. On the other hand, the frequency of severe DKA observed in the present study is four-to-five fold higher than that reported from Finland [28]. The reason for this difference might be that the background higher prevalence of diabetes mellitus in Finland makes the recognition of the disease easier by both the public and the physician, reducing delays in diagnosis and treatment, ultimately preventing severe form of DKA. The older age of our patients might be contributory, given that the frequency of severe form of DKA has been shown to be higher in patients between the age 10–14 years compared to patients aged 5–9 years [21]. Consistent with the report of Habib, [18] majority of the patients with impaired consciousness at presentation in the present study, had severe form of DKA. In that study it was revealed that there was a strong correlation between degree of acidosis and depression of the central nervous system [18].

When compared with reports from developed countries of the west, [2, 4, 30, 31] data from the present study revealed some differences in clinical characteristics of the patients. For instance, the prevalence of DKA at first presentation of T1DM, the mean age at presentation, the mean duration of symptoms before presentation and the mean blood glucose at the point of admission were all higher in the present series, representing some noteworthy features of diabetes in children and adolescents in a developing country. Similarly, reports from the Gulf countries such as Saudi Arabia, Kuwait, United Arab Emirates and Iran are generally in agreement with the findings in the present study [16–18, 26, 32]. The implication is that the clinicians should be alert to these differences depending on where they are practicing. Hypoglycaemia was the leading complication encountered during the management of DKA in this series. It occurred in one-sixth of the cases, indicating the need for clinicians to be alert to its occurrence with the aim of preventing it.

One limitation of the present study need to be considered. The relatively small sample size from one healthcare facility. Future multicentre study with a larger sample size is being designed to enhance the interpretation of results, and ultimately strengthen conclusions. Despite this limitation, the study gave an insight into the clinical characteristics of newly-diagnosed diabetes presenting with DKA in our local community.

## Conclusions

In conclusion, three-quarter of children and adolescents present with DKA as first manifestation of T1DM with no significant decline in frequency over a 16-year period in UBTH.

## Authors’ original submitted files for images

Below are the links to the authors’ original submitted files for images. Authors’ original file for figure 1
