# Supplementary figures and images for: Ketoacidosis at diagnosis of type 1 diabetes in children and adolescents: frequency and clinical characteristics
Source: J Diabetes Metab Disord. 2013 Dec 19;12:47. doi: 10.1186/2251-6581-12-47 (PMC7963434; doi:10.1186/2251-6581-12-47)

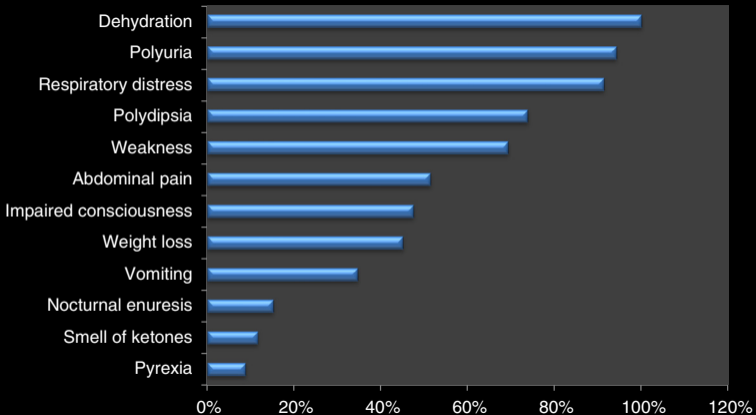

Supplement: Supplementary file 1 — Authors’ original file for figure 1 [file 40200_2012_179_MOESM1_ESM.pdf]
